# Bone mineral density, adiposity, and cognitive functions

**DOI:** 10.3389/fnagi.2015.00016

**Published:** 2015-02-18

**Authors:** Hamid R. Sohrabi, Kristyn A. Bates, Michael Weinborn, Romola S. Bucks, Stephanie R. Rainey-Smith, Mark A. Rodrigues, Sabine M. Bird, Belinda M. Brown, John Beilby, Matthew Howard, Arthur Criddle, Megan Wraith, Kevin Taddei, Georgia Martins, Athena Paton, Tejal Shah, Satvinder S. Dhaliwal, Pankaj D. Mehta, Jonathan K. Foster, Ian J. Martins, Nicola T. Lautenschlager, Francis Mastaglia, Simon M. Laws, Ralph N. Martins

**Affiliations:** ^1^School of Medical Sciences, Edith Cowan UniversityJoondalup, WA, Australia; ^2^The McCusker Alzheimer's Research FoundationNedlands, WA, Australia; ^3^School of Psychiatry and Clinical Neurosciences, University of Western AustraliaCrawley, Australia; ^4^The School of Animal Biology, University of Western AustraliaCrawley, WA, Australia; ^5^School of Psychology, University of Western AustraliaCrawley, WA, Australia; ^6^School of Pathology and Laboratory Medicine, University of Western AustraliaNedlands, WA, Australia; ^7^PathWest Laboratory Medicine of WANedlands, WA, Australia; ^8^Western Medicine, Hollywood Specialist CentreNedlands, WA, Australia; ^9^School of Public Health, Curtin University of TechnologyPerth, WA, Australia; ^10^Division of Immunology, Department of Developmental Neurobiolog,Institute for Basic Research in Developmental DisabilitiesStaten Island, NY, USA; ^11^Neurosciences Unit, Health Department of WA, School of Psychology and Speech Pathology, Curtin University of TechnologyPerth, WA, Australia; ^12^Academic Unit for Psychiatry of Old Age, St. Vincent's Health, Department of Psychiatry, University of MelbourneParkville, VIC, Australia; ^13^The WA Centre for Health and Ageing, University of Western AustraliaCrawley, Australia; ^14^Institute for Immunology and Infectious Diseases, Murdoch UniversityWA, Australia

**Keywords:** dual energy x-ray absorptiometry, cognition, apolipoprotein E, bone mineral density, episodic verbal memory, executive function, aging

## Abstract

Cognitive decline and dementia due to Alzheimer's disease (AD) have been associated with genetic, lifestyle, and environmental factors. A number of potentially modifiable risk factors should be taken into account when preventive or ameliorative interventions targeting dementia and its preclinical stages are investigated. Bone mineral density (BMD) and body composition are two such potentially modifiable risk factors, and their association with cognitive decline was investigated in this study. 164 participants, aged 34–87 years old (62.78 ± 9.27), were recruited for this longitudinal study and underwent cognitive and clinical examinations at baseline and after 3 years. Blood samples were collected for apolipoprotein E (APOE) genotyping and dual energy x-ray absorptiometry (DXA) was conducted at the same day as cognitive assessment. Using hierarchical regression analysis, we found that BMD and lean body mass, as measured using DXA were significant predictors of episodic memory. Age, gender, APOE status, and premorbid IQ were controlled for. Specifically, the List A learning from California Verbal Learning Test was significantly associated with BMD and lean mass both at baseline and at follow up assessment. Our findings indicate that there is a significant association between BMD and lean body mass and episodic verbal learning. While the involvement of modifiable lifestyle factors in human cognitive function has been examined in different studies, there is a need for further research to understand the potential underlying mechanisms.

## Introduction

Dementia is a major debilitating disorder and a cause of significant concern for the currently aging population. In 2010, more than 35.6 million individuals were diagnosed with dementia worldwide (Prince et al., 2013). Prevalence projections indicate that dementia cases will dramatically increase worldwide by 2050 (Norton et al., 2014). In particular, a report from the Australian Institute of Health and Welfare estimates that the number of dementia patients in Australia will increase from 175,000 to 465,000 by the year 2031 (Australian Institute of Health and Welfare, 2007). Preventative research to reduce dementia-related burden is essential to tackle the financial as well as social consequences of this condition. Based on recent modeling reported by Alzheimer's Australia, if the onset of dementia was delayed by 2 years, a reduction of 13% or 398,000 cumulative new cases by 2050 would be achieved. Further, a delay of 5 years would reduce the number of cumulative new cases by 30%, or 935,000 individuals by 2050. Dementia-prevention programs would have a significant economic impact and improve the quality of life for affected individuals and their family (Vickland et al., 2012). Identification of potentially modifiable risk factors, including lifestyle factors, is a promising avenue for facilitating reductions in dementia incidence.

Dementia due to Alzheimer's disease (AD) is the most common form of dementia worldwide (Di Carlo et al., 2012). While the underlying causes of the late-onset form of the disease remain poorly understood, a complex mix of genetic, lifestyle, and hormonal factors is thought to contribute to the cerebral accumulation of a small peptide, beta amyloid (Aβ) (Butterfield et al., 2002; Isacson et al., 2002; Verdile et al., 2004; Wirths et al., 2004), resulting in the formation of extracellular amyloid deposits (Glenner and Wong, 1984; Masters et al., 1985). Research suggests that one third of AD cases are preventable (Norton et al., 2014). Because lifestyle and hormonal factors are potentially modifiable risk factors for AD, they remain a focus of intense research scrutiny. One such hormone-related risk-factor is osteoporosis, which is defined as bone mineral density (BMD) more than 2.5 standard deviations below the mean for healthy adults aged between 20 and 40 years (W.H.O, 1994). The prevalence of osteoporosis increases with age. According to Osteoporosis Australia, 1 in 2 women and 1 in 3 men over the age of 60 will experience an osteoporotic fracture. In addition to age, female sex and menopause-related changes, previous fragility, previous fragility fractures, family history of hip fracture, and the use of oral corticosteroids are also significant risk factors for low BMD (Kanis, 2002; Finkelstein et al., 2008).

Osteoporosis and low BMD (osteopenia) have been associated with cognitive impairment and dementia (Lui et al., 2003; Rothman et al., 2007). BMD is regulated through the brain (Haberland et al., 2001; Karsenty and Oury, 2010), and this may partially explain the underlying relationship between BMD, cognitive dysfunction, and dementia. The brain regions involved in adiposity, (specifically the hypothalamus), also regulate bone remodeling through complicated and slow processes involving hormones including leptin (Haberland et al., 2001; Crockett et al., 2011). Leptin is thought to mediate BMD via binding to relevant receptors in the ventromedial hypothalamus (Haberland et al., 2001; Yang and Barouch, 2007) suggesting that osteoporosis may represent a neuro-skeletal condition (Takeda, 2009). Of note, plasma leptin level has been negatively associated with dementia and AD risk (Lieb et al., 2009). Additionally, the relationship between lower BMD and dementia may be modulated through cumulative exposure to estrogen as it was found in the Framingham Study, that lower femoral neck BMD was associated with a two-fold increase in risk of AD in women, potentially due to estrogen exposure (Tan et al., 2005).

Adiposity or body fat is another potentially modifiable risk factor associated with cognitive decline and dementia; however, research has produced somewhat conflicting results in this area. While most studies have supported a significant association between adiposity and cognitive decline (Luchsinger et al., 2007; Kerwin et al., 2011) other studies have failed to identify a significant relationship between these two on some of the cognitive functions associated with AD, including verbal memory (Wolf et al., 2007). In some studies, adiposity has been associated with cognitive decline only in men (Kanaya et al., 2009), in individuals above 70 years old (Levine and Crimmins, 2012), or in participants below age 70 (Yoon et al., 2012). Adiposity is a risk factor for diabetes, hypertension, and cardiovascular changes; conditions which themselves contribute to significantly increased risk of AD (for a review see: Gustafson and Luchsinger, 2013) and cognitive decline due to vascular pathologies (Gustafson, 2012). For example, it has been suggested that adiposity, as a risk factor for insulin resistance and hyperinsulinemia may increase amyloid deposits in the brain resulting in AD (Luchsinger and Mayeux, 2007). In sum, the available evidence suggests that midlife central obesity plays a significant role in age-related cognitive decline and significantly increases the risk of dementia (Whitmer et al., 2008).

It is important to note that both increased adiposity and osteoporosis have been associated with cardiovascular disease (CVD) (Banks et al., 1994), which is associated with AD-plasma amyloid-β protein (Bates et al., 2009) and has been shown to increase the risk of cognitive decline and dementia (Qiu et al., 2010; Norton et al., 2014). Interestingly, subclinical CVD increases the risk of bone loss and fracture (den Uyl et al., 2011) and BMD has been inversely associated with CVD (Farhat et al., 2007). Further, cardiovascular problems are associated with osteoporosis; moreover, lipid-related problems may play a role in increasing osteoporosis risk (Brown and Sharpless, 2004). Animal models support the association between osteoporosis and atherosclerosis (Parhami et al., 2000; Price et al., 2001). Observational studies have shown that higher atherogenic lipid profile and lipoproteins are inversely associated with bone density (Dimic et al., 2012; Sarkis et al., 2012) but the exact mechanisms underlying this relationship are unclear (Farhat and Cauley, 2008).

Cholesterol metabolism has been linked to apolipoprotein E epsilon 4 allele (APOE ε 4), a major genetic risk factor for late-onset AD (Corder et al., 1993; Saunders et al., 1993; Roses, 1997). The ApoE protein is the major cholesterol transport protein in the brain, with allelic polymorphism in the APOE gene resulting in isoform-specific functional effects (e.g., higher risk of AD for ε 4 carriers and more resistance to AD in ε 2 carriers) (Weisgraber, 1994; Mahley et al., 1996). Some studies have indicated that, in addition to increased risk of AD, APOE can also be involved in osteoporosis through mediating vitamin K transportation and/or inhibition of osteoblast differentiation (Kohlmeier et al., 1996; Parhami et al., 1997). However, a more recent study did not support the involvement of APOE genotype in BMD, increased bone loss, or higher risk of osteoporotic fractures (Schoofs et al., 2004).

The current study evaluated the relationship between adiposity, BMD and subsequent cognitive decline with respect to both screening of functional capacity and, more specifically, verbal episodic memory. We assessed BMD, adiposity and cognition, controlling for the potential effects of age, gender, and possession of the APOE ε 4 allele. Specific hypotheses included: (i) higher BMD would be significantly associated with better current and future cognitive functioning, particularly verbal memory; (ii) higher adiposity and lower lean body mass would be related to current cognitive function and predict subsequent cognitive function.

## Materials and methods

### Study design and cohort selection

One hundred and sixty four participants aged 34–87 years old (62.78 ± 9.27) were recruited from a larger, longitudinal, community-based study (the Western Australia Memory Study), investigating molecular and neuropsychological predictors of cognitive decline within younger and older adults (Clarnette et al., 2001; Sohrabi et al., 2009). The results were analyzed at baseline and after a 3-year follow-up. Participants completed annual blood and cognitive testing using standardized, validated screening and diagnostic measures. Exclusion criteria at the recruitment included: Mini Mental State Examination (MMSE) score ≤ 24 (Folstein et al., 1975); clinically diagnosed dementia; untreated depression [Geriatric Depression Scale (GDS) score ≥ 11]; history of neurological or psychiatric disorders affecting cognitive functions (e.g., stroke, Parkinson's disease, epilepsy, schizophrenia) and difficulty understanding or speaking English.

All participants provided written, informed consent to the study procedures including a body composition/BMD scan, using dual energy x-ray absorptiometry (DXA). Cognitive and clinical assessments, along with venous blood sampling occurred on the same day as DXA was performed. The study was approved by the Human Ethics Committees of Edith Cowan University, University of Western Australia, and Hollywood Private Hospital, Western Australia.

### DXA analysis

The DXA technique quantifies bone mineral content by comparing the attenuation that occurs as a result of absorption of photons at two different energy levels, thereby creating a two dimensional BMD (aBMD) map (Van Loan and Mayclin, 1992). This analysis allows for the separation of body mass into bone, lean, and fat components.

In this study, DXA bone density and body composition scans were conducted on a central, whole body, Norland XR-46, pencil beam scanner using software version 4.1.1. The instrument was calibrated daily using a 77-step calibration standard QC phantom. The mean coefficient of variation (over 5 days) was 0.40 for BMD, 0.20 for lean mass and 0.14 for fat mass. Bone density measures were taken at the spine (L2-L4) and at the hip (femoral neck and total hip). The software generated: (i) *t*-scores, which compared each individual against a group, defined as possessing peak bone mass (i.e., normative data from healthy adults aged 20–40 years); and (ii) *z*-scores which compared an individual with data from their own age group.

Whole body scans were divided into regions of interest including head, chest, midriff, pelvis, and limbs. The measures taken included fat and lean mass (in kg), total body fat percentage, Siri formula, and Brozek formula, for underwater weight equivalents (UWE) (Guerra et al., 2010). To estimate body fat %, body mass density is calculated and converted to body fat %, using the Siri or Brozek equations. These are the most commonly equations available (Guerra et al., 2010).

Most of the scores produced by DXA are highly inter-correlated. In order to address potential multicollinearity, we created composite, sample-based *z* scores derived from the sum of all the *z* scores calculated for lean mass and BMD raw scores, divided by the number of scores. The following raw scores were converted to z scores and divided by six (number of scores) to compute the composite lean mass *z* score: Midriff lean mass + Pelvis lean mass + Left leg lean mass + Right leg lean mass + Left arm lean mass + Right arm lean mass. The bone density composite *z* score was calculated by summing the DXA Spine L2-L4, Femoral Neck, and trochanter computed *z* scores divided by three. We did not use a Fat mass composite *z* score, but instead used the Siri UWE as this score is strongly associated with fat % and other fat mass-related scores derived from DXA.

### Clinical and cognitive measures

Participants completed a comprehensive set of clinical and neuropsychological assessments lasting between 1.5 and 2.5 h and were offered breaks as needed. Depression at baseline was measured using the GDS (Yesavage et al., 1982). Premorbid cognitive ability was assessed using the Cambridge Contextual Reading Test (CCRT) (Beardsall, 1998). General cognitive functioning was assessed using the CAMCOG-R (Roth et al., 1998). Verbal episodic memory was assessed using the California Verbal Learning Test (CVLT) (Delis et al., 1988). Baseline and 3-year follow-up scores for the CVLT were calculated as follows: List Learning (List A; trials 1–5 total score), short delay free recall (SDFR), short delay cued recall (SDCR), long delay free recall (LDFR), long delay cued recall (LDCR), and recognition discriminability (RecDisc).

### Biochemical and genetic analysis

On the same day as the DXA scan and cognitive/clinical assessment, a fasted venous blood sample was collected into serum, EDTA (containing prostaglandin E to prevent platelet activation) and heparin blood collection tubes (Interpath Services, Australia). The whole blood was then separated into different components using standard centrifugation techniques. DNA was isolated from leukocytes, and APOE genotype was determined via polymerase chain reaction (PCR) amplification and restriction enzyme digestion using the method originally described by Hixson and Vernier (1990), and outlined in Laws et al. (2002).

### Statistical analysis

Data were entered into Microsoft Excel and statistical analyses conducted using IBM SPSS Version 19 (IBM SPSS Statistics, 2010 New York, IBM Corp). After testing for normality, descriptive sample characteristics were analyzed. Next, a series of two-step hierarchical linear regressions were conducted in order to explore the relationships between biological variables, general cognitive function (as assessed by the CAMCOG-R) and episodic verbal memory (as measured by the above-mentioned CVLT sub scores). In step one, potential covariates including age at scan time, gender, APOE ε 4 allele status, and premorbid IQ (CCRT) were entered into the analysis. In step 2, variables derived from DXA were entered into the model. These variables included (1) Siri UWE Fat percentage, (2) Lean mass composite score, and (3) BMD composite score.

## Results

In the current study, participants included 69% women and 38% of participants were *APOE* ε 4 carriers. Descriptive data for males/females, and *APOE* ε 4 +/*APOE* ε 4 – groups are presented in Tables 1, 2. The percentage of participants who were *APOE* ε 4 + did not differ by gender, χ^2^(1; *N* = 162) = 0.010, *p* = 0.919. There were significant differences between men and women on most of the DXA measures. The differences are presented in Table 2 with respect to gender and *APOE* ε 4 status. *APOE* ε 4 status and GDS (depression level) were not significantly associated with any of the DXA or cognitive variables.

**Table 1:** **Descriptive findings of dual energy x-ray absorptiometry (DXA) including the *p*-values corresponding to independent *t*^†^**.

	**Sex**			**APOE ε4^e^ Status**		
	**Male (*N* = 51)**	**Female (*N* = 113)**	***P***	**Non-Carrier (*N* = 102)**	**Carrier (*N* = 61)**	***P***
	**Mean (±*SD*)**	**Mean (±*SD*)**		**Mean (±*SD*)**	**Mean (±*SD*)**	
Age at scan	63.65 (±7.88)	62.39 (±9.85)	0.423	63.31 (±9.36)	61.97 (±9.21)	0.372
Total fat %	25.65 (±6.05)	40.27 (±8.07)	0.000^*^	36.63 (±10.36)	34.30 (±9.61)	0.155
Siri UWE fat %	21.14 (±5.67)	32.71 (±7.35)	0.000^*^	29.89 (±8.75)	27.87 (±8.62)	0.153
Lean mass composite score^a^	5.13 (±3.08)	−2.31 (±3.10)	0.000^*^	0.100 (±4.68)	−0.307 (±4.49)	0.586
BMD composite score^b^	0.0007 (±0.944)	−0.0003 (±0.855)	0.999	0.018 (±0.922)	−0.051 (±0.805)	0.626
HDL^c^	1.21 (±0.364)	1.59 (±0.419)	0.000^*^	1.46 (±0.431)	1.50 (±0.458)	0.570
LDL^d^	2.81 (±0.989)	3.13 (±0.851)	0.032^*^	3.01 (±0.878)	3.08 (±0.959)	0.626
Cholesterol	4.93 (±0.992)	5.44 (±0.885)	0.001^*^	5.26 (±0.931)	5.34 (±0.979)	0.584
Triglycerides	1.98 (±1.40)	1.54 (±1.03)	0.027^*^	1.69 (±1.17)	1.64 (±1.19)	0.798

† Equal variances not assumed;

* p < 0.05;

a Lean mass composite score included the DXA Lean mass Z scores for Midriff, Pelvis, Left leg, Right leg, Left arm, and Right arm divided by six;

b Bone Mineral Density Composite Score included the Z scores for DXA Spine L2-L4, Femoral Neck, and trochanter divided by three;

c High-density lipoprotein;

d Low-density lipoprotein;

e *Apolipoprotein E ε4*.

**Table 2:** **Descriptive cognitive data including *p*-values corresponding to independent *t*^†^**.

	**Sex**			**APOE ε4^i^ Status**		
	**Male (*N* = 51)**	**Female (*N* = 113)**	***P***	**Non-Carrier (*N* = 102)**	**Carrier (*N* = 61)**	***P***
	**Mean (±*SD*)**	**Mean (±*SD*)**		**Mean (±*SD*)**	**Mean (±*SD*)**	
CAMCOG^a^-baseline	99.90 (±2.76)	97.79 (±4.40)	0.000^*^	98.72 (±3.50)	97.98 (±4.91)	0.270
CVLT List A^b^-baseline	53.71 (±10.37)	54.61 (±11.20)	0.625	55.05 (±10.83)	52.98 (±11.07)	0.244
CVLT SDFR^c^-baseline	10.75 (±2.86)	11.20 (±2.91)	0.350	10.99 (±2.98)	11.10 (±2.73)	0.817
CVLT SDCR^d^-baseline	11.73 (±2.50)	11.68 (±2.95)	0.926	11.70 (±2.95)	11.62 (±2.54)	0.872
CVLT LDFR^e^-baseline	11.22 (±2.77)	11.17 (±3.04)	0.924	11.32 (±2.92)	10.87 (±2.96)	0.340
CVLT LDCR^f^-baseline	11.61 (±2.81)	11.42 (±3.23)	0.727	11.59 (±3.06)	11.23 (±3.15)	0.475
CVLT RecD^g^-baseline	94.77 (±5.96)	93.26 (±10.76)	0.354	93.70 (±11.11)	93.66 (±6.36)	0.981
CAMCOG-F/U^h^	98.36 (±4.67)	98.12 (±4.23)	0.763	98.30 (±3.96)	97.98 (±5.02)	0.682
CVLT List A-F/U	56.16 (±9.77)	62.09 (±10.41)	0.002^*^	60.41 (±10.39)	59.98 (±11.01)	0.818
CVLT SDFR-F/U	11.30 (±2.89)	12.43 (±2.78)	0.028^*^	11.98 (±3.01)	12.25 (±2.61)	0.588
CVLT SDCR-F/U	12.00 (±2.68)	13.25 (±2.19)	0.004^*^	12.82 (±2.44)	12.92 (±2.41)	0.805
CVLT LDFR-F/U	11.66 (±3.26)	12.87 (±2.52)	0.018^*^	12.58 (±2.57)	12.31 (±3.24)	0.584
CVLT LDCR-F/U	12.07 (±2.98)	13.35 (±2.30)	0.006^*^	13.00 (±2.38)	12.85 (±2.95)	0.736
CVLT RecD-F/U	95.02 (±4.83)	96.04 (±6.95)	0.380	95.83 (±4.86)	95.50 (±8.40)	0.766

† Equal variances not assumed;

* p < 0.05;

a The Cambridge Cognitive Examination-Revised total score;

b The California List A Learning trials 1–5 total score;

c The California Verbal Learning Test (CVLT) Short Delay Free Recall;

d The CVLT Short Delay Cued Recall;

e The CVLT Long Delay Free Recall;

f The CVLT Long Delay Cued Recall;

g CVLT discriminability;

h Follow up;

i *Apolipoprotein E ε4*.

### Correlations

Table 3 presents the correlations between DXA, adiposity, and cognitive measures at baseline and follow up. Premorbid IQ (as measured using the CCRT) was not associated with any of the DXA measures, but was a significant correlate of CAMCOG Baseline and FU scores, and CVLT SDCR and RecD follow up scores. GDS score was also not significantly associated with any of the DXA or other biomarkers and therefore was not included as a covariate in regression analyses. However, we found significant associations between overall cognitive status, as measured by CAMCOG-R, and various DXA measures. A significant, negative association was found between baseline CAMCOG-R and Siri UWE (*r* = −0.204, *p* < 0.01). Lean mass and BMD composite scores were positively associated with verbal learning (CVLT scores) and general cognitive functioning (CAMCOG-R) (Table 3) and therefore included in the subsequent regression analyses. HDL was significantly associated with baseline CVLT subscales including, SDFR (*r* = 0.195, *p* < 0.05), SDCR (*r* = 0.160, *p* < 0.05), and LDCR (*r* = 0.162, *p* < 0.05) but not with the CVLT follow up results except for SDCR (*r* = 0.184, *p* < 0.05). Interestingly, HDL was negatively associated with the total perseveration score for List A learning trials 1–5 (*r* = −0.160, *p* < 0.05), indicating that greater perseveration was associated with lower HDL.

**Table 3:** **Associations between Dual-energy X-ray absorptiometry (DXA), baseline, and follow up cognitive measures (2-tailed)**.

	**Scan age**	**CCRT^a^**	**HDL^b^**	**LDL^c^**	**Cholest^d^**	**Trigl^e^**	**Siri UWE^f^ fat %**	**Lean mass comp^g^**	**BMD comp^h^**	**DXA *t*-score**	**DXA *z*-score**
Scan age		−0.001	−0.083	−0.121	−0.061	0.160^*^	0.077	−0.092	0.067	−0.275^**^	0.009
GDS^i^-baseline	−0.065	−0.074	−0.110	0.061	−0.014	−0.046	0.006	0.094	−0.02	−0.009	−0.003
CAMCOG^j^-baseline	−0.222^**^	0.174^*^	−0.055	−0.008	0.011	0.079	−0.204^**^	0.214^**^	−0.061	−0.028	−0.077
CVLT List A^k^-baseline	−0.206^**^	0.026	0.136	−0.052	0.026	0.035	0.035	−0.028	0.157^*^	0.240^**^	0.155^*^
CVLT-SDFR^l^-baseline	−0.169^*^	0.064	0.195^*^	−0.038	0.03	−0.03	0.055	−0.095	0.049	0.158^*^	0.049
CVLT–SDCR^m^-baseline	−0.057	0.074	0.160^*^	−0.062	0.024	0.017	0.006	−0.054	0.166^*^	0.205^**^	0.160^*^
CVLT-LDFR^n^-baseline	−0.124	0.149	0.147	0.007	0.047	−0.039	−0.016	−0.007	0.078	0.12	0.063
CVLT-LDCR^o^-baseline	−0.164^*^	0.078	0.162^*^	0.043	0.067	−0.076	−0.024	−0.02	0.083	0.14	0.071
CVLT-RecD^p^-baseline	−0.146	−0.058	0.167^*^	−0.015	−0.037	−0.176^*^	−0.029	−0.051	0.066	0.157^*^	0.091
CAMCOG-F/U^q^	−0.218^**^	0.379^**^	0.004	−0.006	0.048	0.094	−0.016	0.054	0.017	0.116	0.041
CVLT List A-F/U	−0.293^**^	0.132	0.155	0.119	0.152	−0.043	0.149	−0.189^*^	0.187^*^	0.319^**^	0.190^*^
CVLT SDFR-F/U	−0.350^**^	0.152	0.166	0.11	0.137	−0.056	0.064	−0.098	0.083	0.233^**^	0.092
CVLT SDCR-F/U	−0.337^**^	0.283^**^	0.184^**^	0.117	0.136	−0.081	0.106	−0.134	0.085	0.186^*^	0.116
CVLT LDFR-F/U	−0.332^**^	0.132	0.087	0.078	0.116	0.013	0.079	−0.098	0.143	0.280^**^	0.159
CVLT LDCR-F/U	−0.343^**^	0.166	0.105	0.026	0.06	−0.009	0.099	−0.12	0.104	0.286^**^	0.152
CVLT RecD-F/U	−0.311^**^	0.266^**^	−0.021	0.013	0.023	0.041	0.092	0.002	0.068	0.169^*^	0.083

* p < 0.05;

** p < 0.011;

a Cambridge Contextual Reading Test;

b High-density lipoprotein;

c Low-density lipoprotein;

d Cholesterol;

e Triglyceride;

f Underwater weight equivalents;

g Lean mass composite score included the DXA Lean mass Z scores for Midriff, Pelvis, Left leg, Right leg, Left arm, and Right arm divided by six;

h Bone Mineral Density Composite Score included the Z scores for DXA Spine L2-L4, Femoral Neck, and trochanter divided by three;

i Geriatric Depression Scale;

j The Cambridge Cognitive Examination-Revised total score;

k The California Verbal Learning Test (CVLT) List A Learning trials 1–5 total score;

l The CVLT Short Delay Free Recall;

m The CVLT Short Delay Cued Recall;

n The CVLT Long Delay Free Recall;

o The CVLT Long Delay Cued Recall;

p The CVLT discriminability;

q *Follow up*.

### Clinical and cognitive data

Clinical and cognitive data were available for all participants. The mean clinical and cognitive scores for all participants are outlined in Tables 1, 2. The means for all neuropsychological measures were within expected age-related norms (Yesavage et al., 1982; Delis et al., 2000). We did not find any significant differences between *APOE* ε 4 carriers and non-carriers on any of the DXA or neuropsychological measures. Depression was not significantly associated with any of the DXA measures (Table 3) or with cognitive measures at baseline or follow up.

### Regression analysis

A series of hierarchical multiple regressions was conducted to examine the associations between DXA and adiposity measures and cognitive test results whilst controlling for the effects of relevant covariates at both baseline and follow up assessments. As discussed, demographic factors including age, gender, APOE ε 4 carriage, and premorbid IQ (as measured using the CCRT) were entered in Step 1. In Step 2, variables derived from DXA were entered into the model including the Siri UWE Fat percentage, Lean mass composite score, and the BMD composite score.

### Cognitive results

The DXA scores in our two-step hierarchical regression model were not significantly associated with general cognitive functioning at baseline (*R*^2^-change = 0.007; *F* change = 0.359, *p* = 0.782) or follow up (*R*^2^-change = 0.002; *F* change = 0.110, *p* = 0.954). In predicting baseline CVLT List A trials 1–5, the first model containing covariates only was not significant [*F*_(4, 141)_ = 2.014, *p* = 0.096, *R*^2^ = 0.054]. However, the second model was significant [*F*_(7, 138)_ = 2.359, *p* < 0.05, *R*^2^ = 0.107]. Specifically, as seen in Table 4, the addition of the DXA variables provided unique predictive variance (*R*^2^-change = 0.53). Higher BMD was significantly associated with higher learning scores, and there was a trend for lower Lean mass composite score (*B* = −0.644; *t* = −1.926; *p* = 0.056).

**Table 4:** **Hierarchical linear regression predicting baseline CVLT List A trials 1–5 total score from lean mass and bone density composite scores**.

	***B***	**Std.error**	**β**	**Adj. *R*^2^**	**Δ*R*^2^**
Step 1				0.027	0.054
Age	−0.247	0.097	−0.209^*^		
Gender	0.558	1.931	0.024		
APOE ε4 status	−2.413	1.849	−0.107		
CCRT^a^	0.062	0.185	0.028		
Step 2				0.062	0.053^*^
Age	−0.322	0.101	−0.272^**^		
Gender	−5.201	3.629	−0.221		
APOE ε4 Status	−2.457	1.837	−0.109		
CCRT	0.073	0.183	0.032		
Siri UWE^b^ fat %	0.074	0.132	0.059		
Lean mass comp score^c^	−0.644	0.334	−0.274^#^		
BMD comp score^d^	3.123	1.138	0.252^**^		

* p < 0.05;

** p < 0.01;

# p < 0.10 (trend);

a Cambridge Contextual Reading Test;

b Underwater weight equivalents;

c Lean mass composite score included the DXA Lean mass Z scores for Midriff, Pelvis, Left leg, Right leg, Left arm, and Right arm divided by six;

d *Bone Mineral Density Composite Score included the Z scores for DXA Spine L2-L4, Femoral Neck, and trochanter divided by three*.

Baseline CVLT discriminability (recognition) performance was not significantly predicted by the first model containing covariates only [*F*_(4, 140)_ = 1.115, *p* = 0.352, *R*^2^ = 0.031, but the second model was significant, *F*_(7, 137)_ = 2.422, *p* < 0.05, *R*^2^ = 0.110]. Both lean mass composite score and BMD were significant predictors of RecDisc in the final model, with higher BMD and lower lean mass being associated with better performance (Table 5). Adding DXA variables contributed an additional 8% of predictive variance.

**Table 5:** **Hierarchical linear regression predicting baseline CVLT discriminability score from lean mass and bone density composite scores**.

	***B***	**Std.error**	**β**	**Adj. *R*^2^**	**Δ*R*^2^**
Step 1				0.003	0.031
Age	−0.155	0.086	−0.150		
Gender	−1.685	1.715	−0.082		
APOE ε4 status	−0.238	1.641	−0.012		
CCRT^a^	−0.113	0.165	−0.057		
Step 2				0.065	0.079^**^
Age	−0.251	0.088	−0.243^**^		
Gender	−10.253	3.178	−0.499^**^		
APOE ε4 status	−0.460	1.609	−0.023		
CCRT	−0.089	0.160	−0.045		
Siri UWE^b^ fat %	0.104	0.115	0.095		
Lean mass comp score^c^	−0.970	0.293	−0.471^***^		
BMD comp score^d^	2.409	0.996	0.222^*^		

* p < 0.05;

** p < 0.01;

*** p < 0.001;

a Cambridge Contextual Reading Test;

b Underwater weight equivalents;

c Lean mass composite score included the DXA Lean mass Z scores for Midriff, Pelvis, Left leg, Right leg, Left arm, and Right arm divided by six;

d *Bone Mineral Density Composite Score included the Z scores for DXA Spine L2-L4, Femoral Neck, and trochanter divided by three*.

CVLT List A learning at follow-up was also significantly predicted by DXA variables. The first model containing covariates only was significant [*F*_(4, 124)_ = 6.005, *p* < 0.001]. The second model was also significant [*F*_(7, 121)_ = 5.375, *p* < 0.001, *R*^2^ = 0.237]. Specifically, the Lean mass composite score (*t* = −2.297, *p* < 0.05), and BMD composite score (*t* = 3.351, *p* < 0.001) were significant predictors (Table 6), accounting for an additional 8% of variance.

**Table 6:** **Hierarchical linear regression predicting follow up CVLT List A trials 1–5 total score from lean mass and bone density composite scores**.

	***B***	**Std.error**	**β**	**Adj. *R*^2^**	**Δ*R*^2^**
Step 1				0.135	0.162^***^
Age	−0.318	0.094	−0.279^***^		
Gender	5.476	1.872	0.241^**^		
APOE ε4 Status	−0.885	1.792	−0.041		
CCRT^a^	0.279	0.180	0.128		
Step 2				0.193	0.075^**^
Age	−0.400	0.096	−0.351^***^		
Gender	−0.665	3.459	−0.029		
APOE ε4 status	−0.993	1.751	−0.046		
CCRT	0.294	0.174	0.135		
Siri UWE^b^ fat %	0.050	0.125	0.041		
Lean mass comp score^c^	−0.732	0.319	−0.322^*^		
BMD comp score^d^	3.633	1.084	0.304^***^		

* p < 0.05;

a p < 0.01;

*** p < 0.001;

a Cambridge Contextual Reading Test;

b Underwater weight equivalents;

c Lean mass composite score included the DXA Lean mass Z scores for Midriff, Pelvis, Left leg, Right leg, Left arm, and Right arm divided by six;

d *Bone Mineral Density Composite Score included the Z scores for DXA Spine L2-L4, Femoral Neck, and trochanter divided by three*.

Due to significant differences between men and women on DXA measures, we examined the interaction of gender and DXA measures (i.e., Siri UWE Fat %, lean mass composite score, and BMD composite score) in predicting cognitive functions. The cognitive functions examined here included baseline and follow up CAMCOG-R, List A learning trials 1–5, SDFR, SDCR, LDCR, LDFR, and Recognition Disc results. Interestingly, we did not find any significant results for these interactions except for Gender *X* BMD on the follow up LDFR [*R*^2^-change = 0.052; *F* change = 2.707, *p* = 0.048; *F*_(10, 118)_ = 3.944, *p* < 0.001; *B* = −1.302, *SE* = 0.606, Beta = −0.698; *t* = −2.150, *p* < 0.034]. Further analysis indicated that higher BMD was significantly associated with better follow up LDFR in men, but not in women (*r* = 0.459, *p* < 0.002 and *r* = −0.039, *p* = 0.707, respectively).

## Discussion

The results of this longitudinal study lend further support to the mounting body of evidence suggesting that lifestyle factors may be involved in cognitive functions and, in turn, may modulate the risk of future cognitive decline. We report a significant link between potentially modifiable indicators of systemic health, namely BMD and Lean body mass on cognitive performance in a group of community-dwelling, healthy adults. These findings have a number of potential implications for understanding the underlying, modifiable mechanisms involved in pathological cognitive decline and for developing preventive and potentially ameliorative clinical trials targeting AD, specifically with respect to the possible influence of metabolic, cardiovascular, and general health factors.

BMD was a predictor for cognitive performance with respect to CVLT measures (Tables 4–6) in a cross-sectional as well as longitudinal context. Interestingly, the findings from the present study indicate that the effect of *APOE* ε4 was limited, i.e., we did not observe any significant differences between the *APOE* ε4 carriers and non-carriers in the study on DXA or cognitive assessment results (Table 1).

In this study, we found significant differences between men and women on BMD and body composition (as expected from previous studies), but we did not find significant differences on cognitive measures at baseline. However, after 3 years of follow up, women outperformed men on most of the CVLT measures (Table 2) but their performance was similar on follow up global cognitive function. This may be consistent with findings implying differential cognitive decline rate in women vs. men (Maylor et al., 2007; Holland et al., 2013).

Low BMD and osteoporosis restrict morbidity amongst the elderly and have been associated to the risk of future cognitive decline (Yaffe et al., 1999; Lui et al., 2003) and dementia due to AD (Tan et al., 2005). Low BMD has been associated with lower cognitive function in cross-sectional study of post-menopausal women (Brownbill and Ilich, 2004) and with verbal memory performance in both men and women (Zhang et al., 2001). It has been suggested that BMD may be reflective of cumulative estrogen exposure, thus providing a molecular mechanism for the link between BMD and cognition as longer estrogen exposure has been associated with lower dementia risk (Fox et al., 2013). However, the evidence supporting a role for estrogen replacement in protecting against AD is currently uncertain due to a number of methodological factors and possible confounding variables including education, general health and physical activity (as reviewed in: Yaffe et al., 1998). As such, the debate surrounding the efficacy of estrogen replacement as a therapeutic target for AD continues.

We found that BMD and lean mass were significantly associated with memory abilities as measured using the CVLT (Tables 4–6). This is consistent with a previous report that BMD and verbal memory impairment were significantly associated (Zhang et al., 2001). Zhang et al. (2001) has postulated that this relationship may be potentially a function of cumulative exposure to estrogen. In fact, our findings, indirectly, support the notion of BMD as a marker of cumulative estrogen exposure, particularly because we observed a significant association between sex and CVLT Learning abilities at follow-up in the present study (Table 2). It should be noted that we did not measure estrogen exposure in this study. However, it has previously been shown that body fat serves as a source of endogenous estrogen in post-menopausal women (Bagger et al., 2004).

The relationship between memory, BMD, and body composition were more significantly pronounced on performance in the List A learning trials. In the current study, BMD and lean mass measures of DXA were significant predictors of CVLT scores representing learning capabilities. This finding is important, as encoding deficits in episodic verbal memory i.e., mental representation of new information–are the primary memory problems seen in preclinical dementia and AD (Golby et al., 2005; Twamley et al., 2006; Beck et al., 2012), and such encoding problems have been shown to correlate with cholinergic deficits that are commonly seen in AD (White and Ruske, 2002). Interestingly, the cholinergic system plays a pivotal role in BMD regulation, as has recently been reviewed (Eimar et al., 2013). These considerations are consistent with our findings discussed earlier concerning the association between learning ability and BMD.

It has been reported that executive functions are associated with episodic memory (Duff et al., 2005). More specifically, the attention component of executive function plays a significant role in verbal learning and memory (Brooks et al., 2006) and has been associated with difficulties in CVLT List A learning (Hill et al., 2012). Our findings show a significant association between List A learning and BMD (as discussed earlier). This may indirectly suggest an underlying mechanism involved in both BMD and executive functioning, although this has to be carefully examined in future studies.

While the current study furthers our understanding of the relationship between age-related memory capacities and BMD, the cohort size and the very wide age range represent the main limitations. In addition, it would be advantageous to have baseline and follow up DXA results, and general morbidity and physical activity to examine the potential differences with negative changes in the DXA results in terms of memory and other cognitive functions. Additionally, examining the sex hormones will add value to a longitudinal study investigating the relationship between DXA scores and cognitive functions.

Further research is warranted investigating the relationship between BMD and cognitive dysfunctions in various dementia patient groups and in preclinical stages, and the potential mechanisms underlying these relationships. Such research could significantly improve our knowledge of the association between bone mineral content and higher cortical capabilities, and the relevance of these factors in aging and dementia. Future research may investigate the relationship between BMD and cognitive decline in aging while controlling for the effects of general mobility and current/previous physical activity as contributing factors.

The non-significant relationship between *APOE* alleles carriage, BMD and cognition in this study may indicate differential contributing pathways for genetic vs. lifestyle factors. Of course, we requires further research in a larger cohort where various genes identified as contributing to dementia risk can be examined in relation to BMD and cognitive decline measures.

## Author contributions

Study design: HRS, KAB, JKF, SML, and RNM. Study conduct: HRS, KAB, MAR, MH, KT, GM, AP, JKF. Data collection: HRS, KAB, MAR, AP, GM. Data analysis: HRS, KAB, SSD, MW. Data interpretation: HRS, KAB, TS, RSB. Drafting manuscript: HRS, KAB. Revising manuscript content: MW, RSB, SRRS, JB, AC, MW, BMB, SMB, KT, TS, PDM, JKF, IJM, NLL, FM, SML. Approving final version of manuscript: HRS, KAB, MW, SSD, FM, NLL, and RNM. HRS, SSD, MW, and RNM take responsibility for the integrity of the data analysis.

### Conflict of interest statement

RNM is the founder and owns stock in Alzhyme. HRS has received personal compensation for activities with Pfizer and Wyeth and currently with Takeda Pharmaceuticals. The authors declare that the research was conducted in the absence of any commercial or financial relationships that could be construed as a potential conflict of interest.
